# CADASIL vs. Multiple Sclerosis: Is It Misdiagnosis or Concomitant? A Case Series

**DOI:** 10.3389/fneur.2020.00860

**Published:** 2020-09-04

**Authors:** Ayesha Khan, Vida Abedi, Jiang Li, Muhammad T. Malik, Megan Esch, Ramin Zand

**Affiliations:** ^1^Geisinger Neuroscience Institute, Geisinger Health System, Danville, PA, United States; ^2^Department of Molecular and Functional Genomics, Weis Center for Research, Geisinger Health System, Danville, PA, United States; ^3^Biocomplexity Institute, Virginia Tech, Blacksburg, VA, United States; ^4^Department of Neurology, University of Tennessee Health Science Center, Memphis, TN, United States

**Keywords:** CADASIL, multiple sclerosis, *NOTCH3*, case series, autoimmune diseases, diagnostic errors, associated conditions

## Abstract

**Introduction:** Cerebral autosomal dominant arteriopathy and subcortical infarct leukoencephalopathy (CADASIL) is the most common form of hereditary stroke caused by a mutation in the *NOTCH3* gene located on the short arm of chromosome 19. A small number of published reports describe CADASIL patients who were initially diagnosed as multiple sclerosis. Although it was previously indicated that there was no association between *NOTCH3* mutations and multiple sclerosis, the involvement of autoimmune mechanisms among patients with CADASIL has been hypothesized.

**Case Presentation:** Case 1 is a middle-aged woman with initial diagnoses of multiple sclerosis (MS) and myelitis that continued to progress despite treatment with disease-modifying agents. She had occasional migraines, transient blurred vision, and multiple lacunar infarcts. She continued treatment for about 15 years with no significant alleviation and progressive changes on brain MRI; genetic testing was ordered which showed *NOTCH3* mutation, and diagnosis was changed to CADASIL with subsequent revision of treatment course. However, the presence of myelitis in this patient is unusual and may raise the question of a concurrent autoimmune process. Case 2 is a woman presenting with vertigo and paresthesia and diagnosed with MS based on an initial brain MRI showing biventricular white matter hyperintensities; however, she was not started on any disease-modifying agents. Her symptoms were reevaluated by a neurologist, and genetic testing was performed for *NOTCH 3*. Case 3 is a young woman with a history of migraines who initially presented with numbness and gait ataxia which later progressed to speech difficulty and memory loss. A diagnosis of MS was established which was later changed to CADASIL.

**Conclusion:** Since CADASIL is a rare disease, it is imperative to raise awareness of its unique clinical condition as well as variation in its clinical presentations. It is crucial that the overlapping symptoms between MS and CADASIL be thoroughly examined to avoid misdiagnosis and treatment complications. The involvement of autoimmune mechanisms in CADASIL and the role of *NOTCH3* gene mutations in provoking an autoimmune process should be further investigated.

## Introduction

Cerebral autosomal dominant arteriopathy and subcortical infarct leukoencephalopathy (CADASIL) is the most common form of hereditary stroke. CADASIL is a genetic disorder caused by cysteine altering mutation in epidermal growth factor-like repeat (EGFr) domain of the *NOTCH3* gene located on chromosome 19q12 (1). The core features of CADASIL are migraine, strokes, dementia, and psychiatric features (2). While typical radiographic features of CADASIL include a predominance of T2 hyperintensities in the bilateral temporal lobes and external capsules, the presence of confluent periventricular hyperintensities or other atypical features (e.g., spinal cord involvement) may mimic other diagnoses including multiple sclerosis (MS).

There have been a small number of published reports describing cases of CADASIL who were initially diagnosed as MS (3–5). Although it was previously indicated that there was no association between *NOTCH3* mutations and MS (6), the involvement of autoimmune mechanisms in some patients with CADASIL has been hypothesized (7, 8). Here, we present a series of three independent cases, where the patients' journey is summarized from the time of initial presentation and diagnosis of MS until the time when CADASIL diagnosis was confirmed. The cases presented in this series were deidentified and reviewed by the privacy office at Geisinger Medical Center to meet the Health Insurance Portability and Accountability Act (HIPAA) Safe Harbor requirements. The Institutional Review Process was not required for publication.

## Case 1

**Figure 1:**
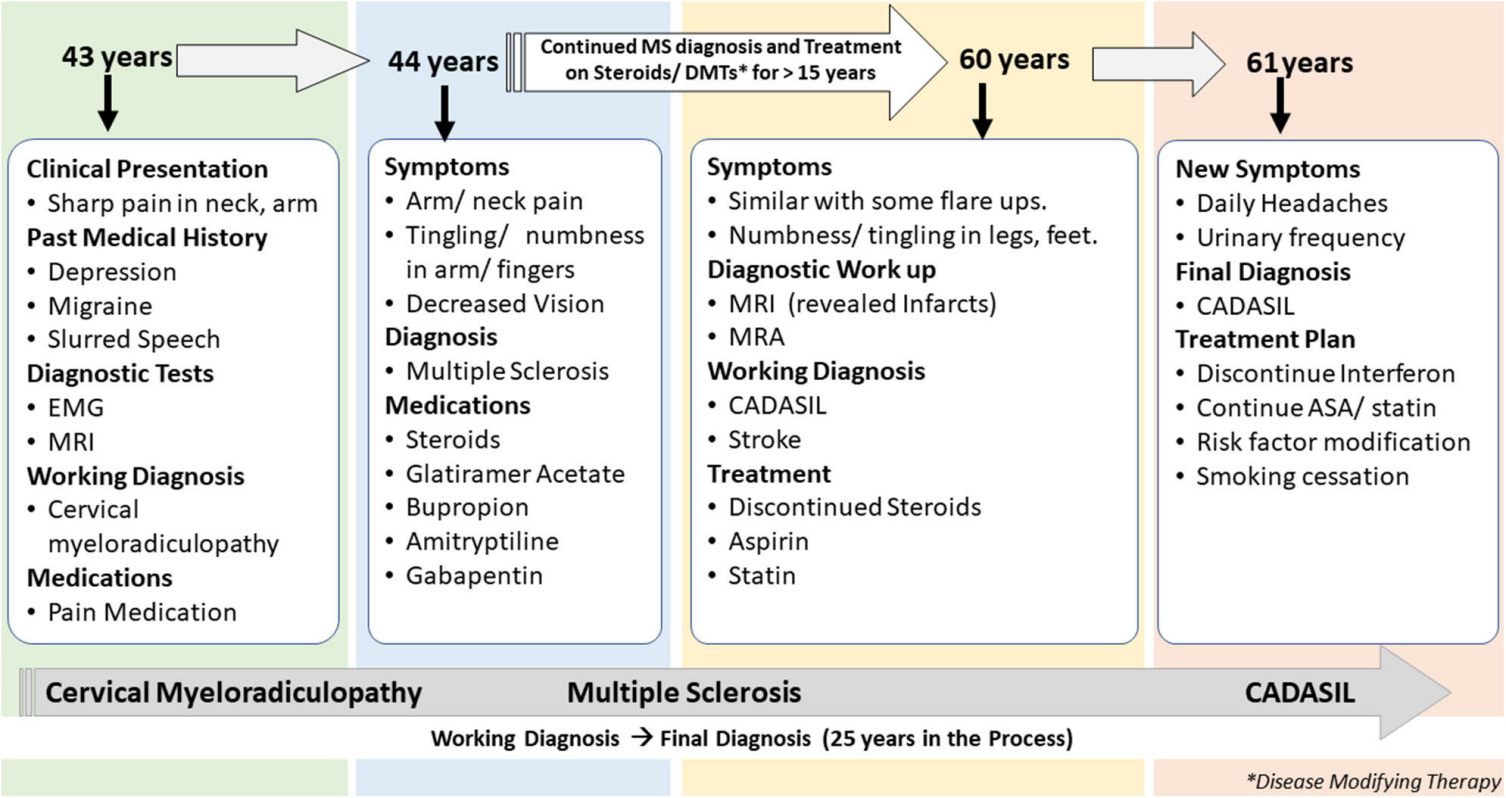
Summary of presentation and diagnosis for Case 1.

The first patient is a 61-years-old woman with a history of migraine headaches, depression treated with paroxetine, memory loss, and paresthesia in her hands and feet for several years. This patient is an active patient at our center and continues to follow up with the neurologist at the age of 61 years. The patient's data were reviewed for the past 19 years when she was first diagnosed with MS (Figure 1). The clinical data included over 30 encounters, office visits, pharmacy notes, and radiology reports among others. She was first referred to a neurologist at the age of 43 years with the complaint of sudden onset of pain in the right shoulder while lifting heavy weight at work. Her past medical history was positive for depression treated with paroxetine, intermittent blurred vision, slurred speech, and occasional episodes of imbalance. The family history included a stroke in her father at 40 years and a brother who died from a stroke at an unknown age. A living brother and son endorsed migraine headaches. Cervical spine imaging demonstrated a C1 dorsal signal abnormality contiguous with the medulla. Degenerative changes were noticed in the cervical spine at C5-6 and C6-7 with spurring and some left foraminal stenosis. Electromyography (EMG) was performed that showed subtle findings of denervation of right-hand muscles. An initial diagnosis of cervical myeloradiculopathy was considered secondary to her shoulder injury, and she was prescribed pain medication for symptom relief. Her symptoms continued to deteriorate with increasing pain in both arms and finger paresthesia. An MRI of the brain demonstrated confluent T2 hyperintensities in the temporal lobe, lenticulostriate nucleus, thalamus, and the brainstem for possible MS. A lumbar puncture was completed in an outside facility, reportedly unremarkable, though the results were unavailable for confirmation. She completed a 2-weeks course of oral prednisone and was started on glatiramer acetate for a presumed diagnosis of MS.

The patient continued to follow up with the neurologist for several years, with a diagnosis of MS. Her presumed MS flares included transient diplopia and intermittent vertigo. Repeat brain MRI showed worsening of T2 hyperintensities within the deep white matter and brain stem. MRI of the cervical spine revealed an anterior T2 hyperintense signal from C4 to C6 and a significant but stable C5 disk bulge. Due to her progressive symptoms of intermittent numbness, burning feet, difficulty with memory, fatigue, increased urinary frequency, and urgency, she was given a diagnosis of secondary progressive MS. Several immunomodulating therapies were tried, including glatiramer acetate, interferon-beta, peginterferon beta-1a, natalizumab, and teriflunomide; she continued to progress.

**Figure 2:**
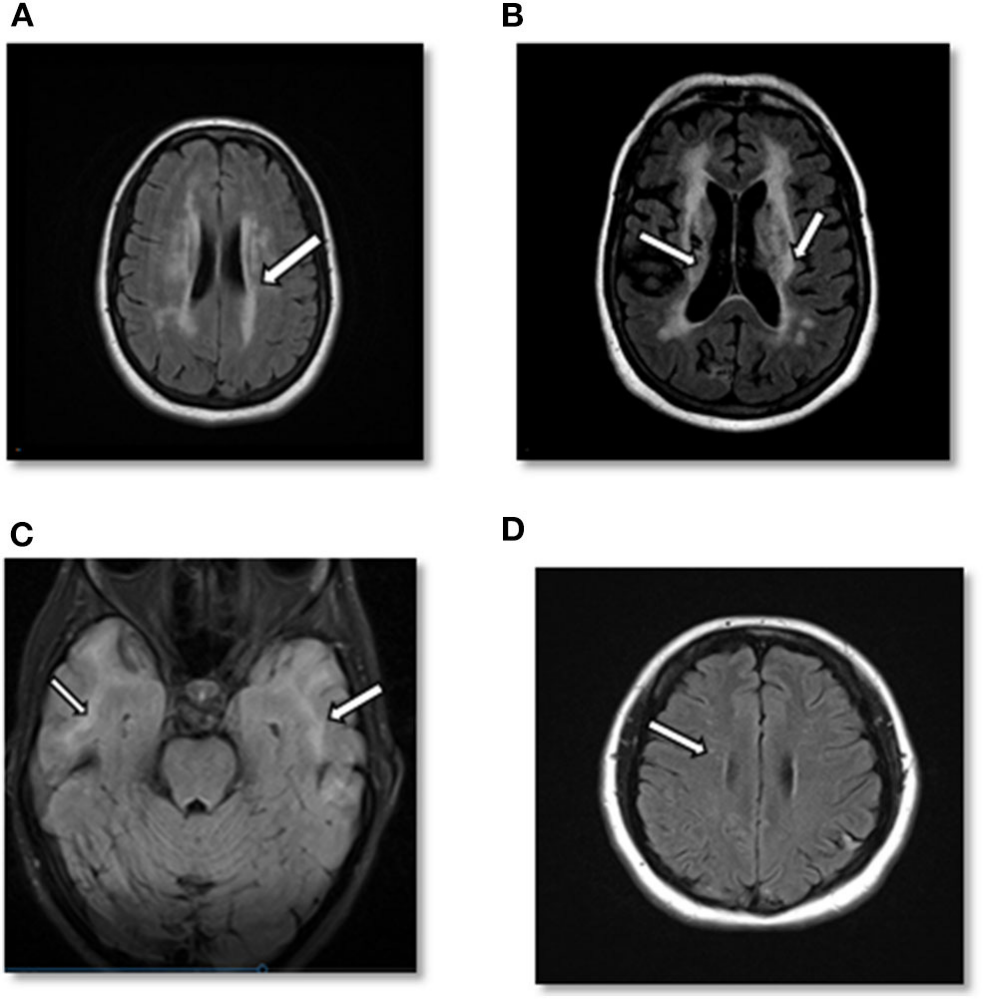
**(A)** Case 1: Axial cerebral T2-FLAIR image demonstrates T2 hyperintense signal in periventricular area. **(B)** Case 1: Axial T2-FLAIR image after 10 years demonstrates significant worsening of T2 hyperintense signal in periventricular and subcortical areas with multiple prominent lacunar infarcts as well as cortical atrophy. **(C)** Case 2: Axial cerebral T2-FLAIR showing confluent white matter hyperintensities in bilateral temporal lobes. **(D)** Case 3: Axial cerebral T2-FLAIR: small foci of T2-FLAIR hyperintensity in the frontal lobe.

At 60 years, a brain MRI showed stable imaging sequelae of T2 hyperintensities with the evolution of right occipital encephalomalacia. MRI of the cervical spine showed stable dorsal signal abnormality at the C1 level and similar multilevel degenerative changes. The MRI was repeated at 61 years and demonstrated multiple lacunar infarcts (Figures 2A,B). The diagnosis was subsequently reevaluated. The imaging findings, along with the clinical picture of recurrent neurological events, and long-standing personal in addition to the family history of stroke and migraine headaches, were suggestive of CADASIL. The patient started a high-dose statin and aspirin therapy. Genetic testing for *NOTCH3* gene mutation was positive revealing a heterozygous missense mutation (NM_00435.2:c.544>T[pArg182Cys]) (9, 10).

## Case 2

**Figure 3:**
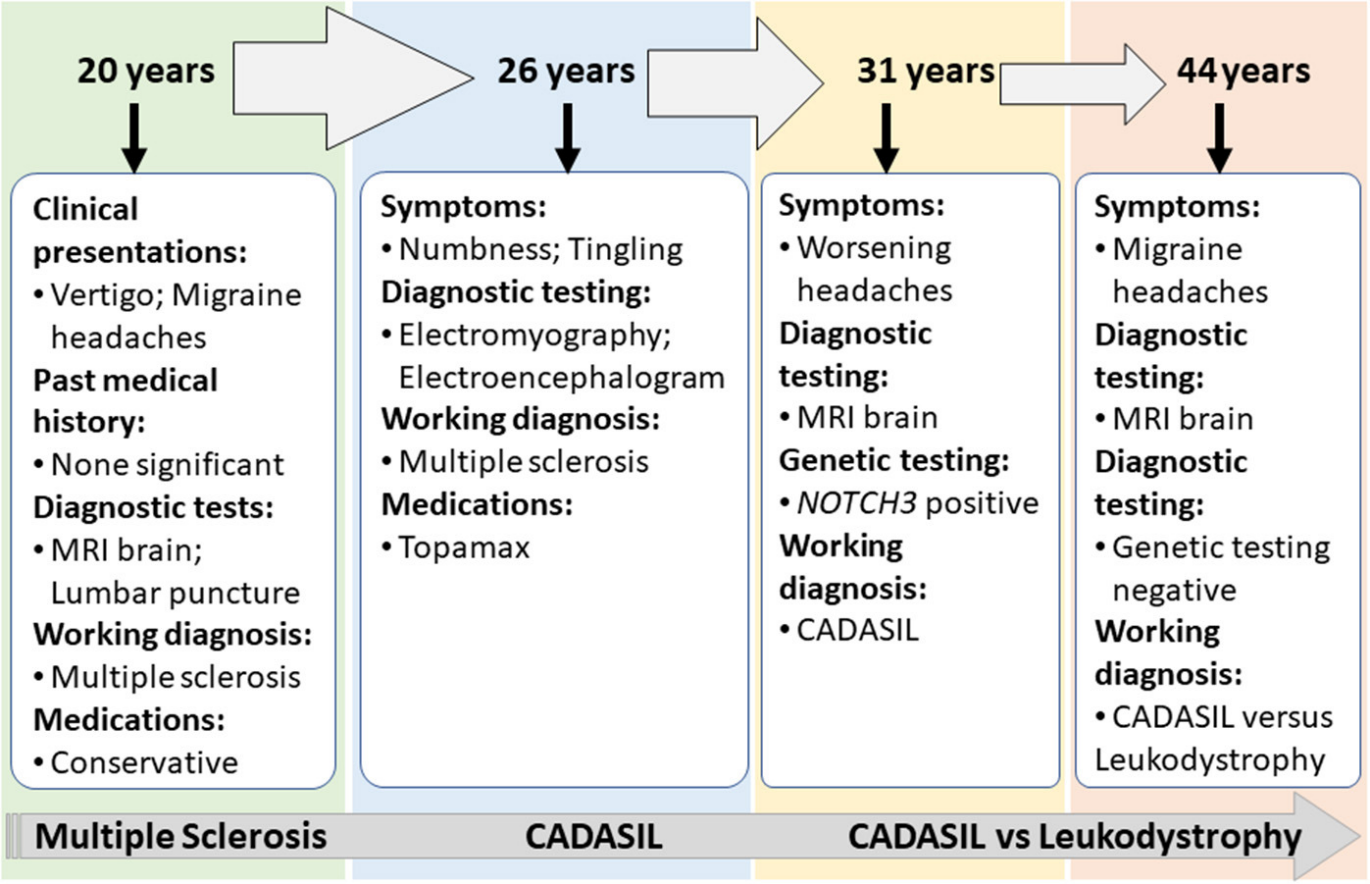
Summary of clinical presentations and diagnosis for Case 2.

Case 2 is a 55-years-old woman who initially presented to an outside hospital with vertigo 20 years prior to being evaluated by a neuroimmunologist. Her symptoms started suddenly at work and gradually worsened. An initial brain MRI demonstrated confluent biventricular periventricular and juxta cortical T2 hyperintensities. Cerebral spinal fluid analysis was within normal limits without any oligoclonal bands. Her initial complaint included numbness and tingling in her hands and feet. However, she was not started on any treatment, and her symptoms resolved after a few months. About a year later, she developed bilateral migraine headaches without aura lasting from days to weeks, associated with nausea, photophobia, and phonophobia. The headache was refractory to typical migraine medications. In the light of the previous abnormal MRI, she underwent brainstem auditory-visual evoked potentials and somatosensory evoked potentials. The results were within normal limits. EMG was done due to concern for possible chronic inflammatory demyelinating polyneuropathy due to worsening numbness and tingling but showed negative results. Fifteen years after the initial episode, the patient started complaining of mild memory loss, increased numbness in hands and feet, and worsening migraine headaches (Figure 3). Her past medical history at that time was significant for generalized fatigue, hypersomnolence, anxiety, and depression. The family history was unremarkable. The diagnosis of MS was made by a general neurologist, and she was referred to an MS clinic. However, an MRI of the brain was repeated at age 55 years which showed a significant worsening of the white matter hyperintensity with the involvement of bilateral temporal and external capsules (Figure 2C). The patient diagnosis was changed to CADASIL based on the new imaging findings and a positive *NOTCH3* genetic test.

## Case 3

Case 3 is a 45-years-old woman with an initial diagnosis of MS who was referred to our center to establish care. She initially developed bilateral lower extremity numbness and impaired balance at age 21. Her past medical history was significant for oral contraceptive pills (OCPs) since the age of 17 and cigarette smoking. Due to the mild symptoms when first evaluated, she was not started on any disease-modifying therapy and was managed conservatively. Several years later in her mid-30s, she saw a neurologist secondary to experiencing word-finding difficulty, short-term memory loss, and difficulty walking. Her past medical history was remarkable for migraine, depression, mild short-term memory loss, ataxia, restless leg syndrome, urinary urgency, and stroke. She was on a 3-months course of glucocorticoids for her MS symptoms at that time. The family history was significant for migraine headaches. She underwent a lumbar puncture at a different center, which was reportedly positive for MS; however, the report of cerebrospinal fluid (CSF) study was not available to us. In addition, an MRI of the brain demonstrated periventricular and subcortical white matter hyperintensities in the frontal and parietal lobes.

**Figure 4:**
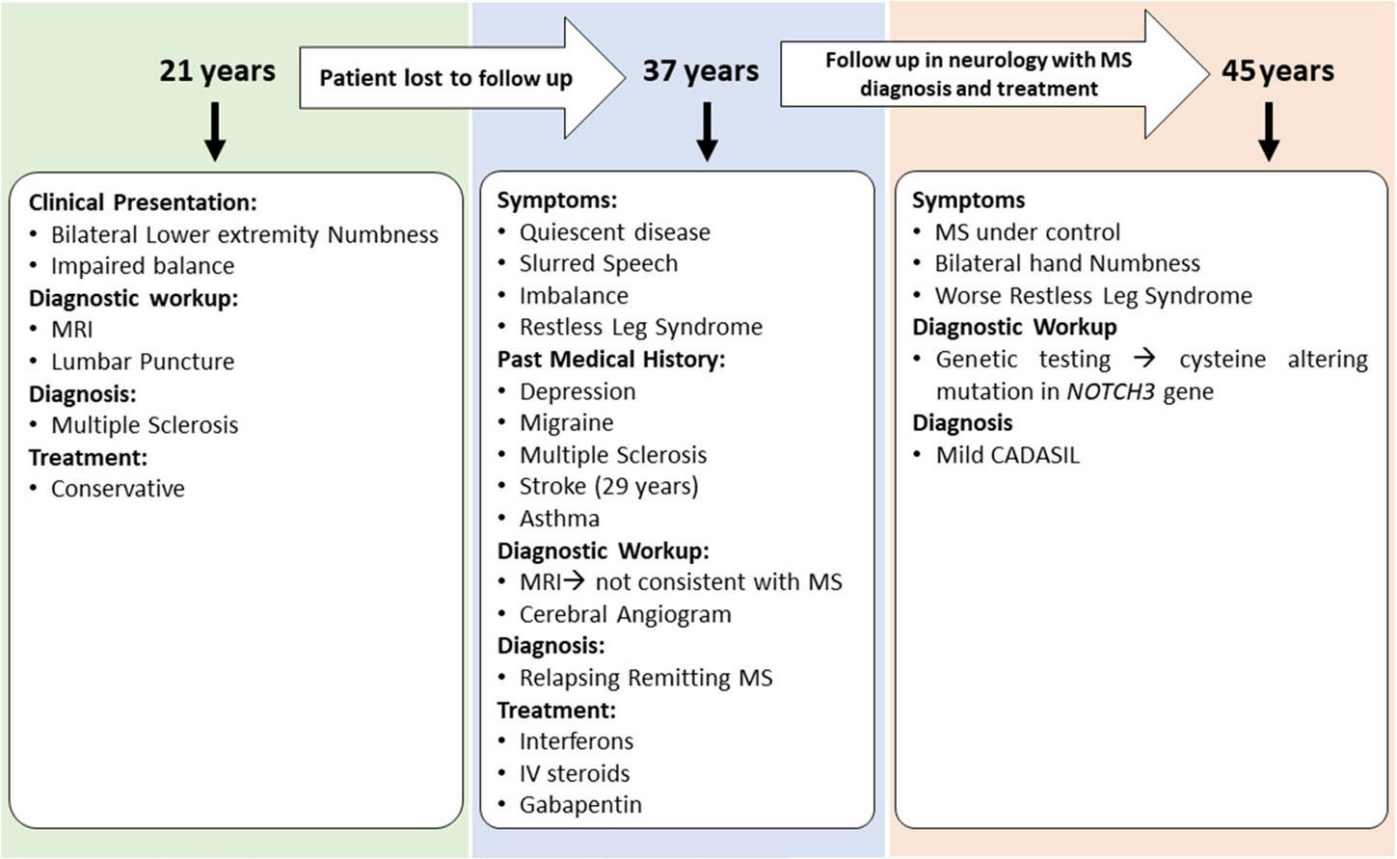
Summary of clinical presentations and diagnosis for Case 3.

She was started on short-term intravenous steroids. A few years later, she returned with several new neurological deficits including numbness and dysesthesias around the mouth, right leg weakness, and right foot drop which were thought to be MS relapses. At that time, the brain MRI demonstrated periventricular and subcortical white matter T2 hyperintense lesions in the frontal and parietal lobes (Figure 2D). The clinicians diagnosed her as having an aggressive form of relapsing-remitting MS and decided to start dimethyl fumarate (disease-modifying therapy). Reviews of the MRI at some later visits suggested findings inconsistent with MS and more suggestive of a vasculopathy. She was referred to a vascular neurologist, who performed a cerebral angiogram that was negative for vasculopathy. Shortly thereafter, she developed a deep vein thrombosis requiring a workup of coagulation profile that revealed mild elevation of factor VIII and fibrinogen. She was started on rivaroxaban and advised to stop OCPs. She continued to be stable on dimethyl fumarate with no MRI changes and did not report any side effects from the medications. After almost 6 years of being on dimethyl fumarate and no significant improvement, she was evaluated by another vascular neurologist for a second opinion. The genetic testing revealed that the patient had a cysteine altering mutation in higher domains of the *NOTCH3* gene (NM_000435.2:c.3691C>T [p.Arg1231Cys]) that causes a milder form of CADASIL (Figure 4) (11, 12). The patient was taken off the disease-modifying agent and continued to follow up with the stroke neurologist and immunologist for symptomatic treatment and management of her new diagnosis.

## Discussion

CADASIL diagnosis can be difficult as the disease prevalence is extremely low for severe cases (about 2 per 100,000), and there is a poor genotype-phenotype correlation (13–15). The heterogeneity of symptoms and the age of onset also contribute to CADASIL being underdiagnosed. Our patients were women in the third and fourth decades of life who initially presented with multiple episodes of neurological dysfunction and T2-MRI changes suggestive of demyelination (Table 1). Although each patient had some red flags at the initial presentation, none of them fulfill the criteria developed by Pescini et al. for CADASIL diagnosis (16). These cases highlight the pitfalls associated with diagnosing patients whose demographics and clinical picture may be more consistent with a common neurological disorder.

**Table 1:** Clinical presentation and similarities in case series.

**Clinical findings**	**Case 1**	**Case 2**	**Case 3**
Age at first presentation	43	35	21
Spinal hyperintensities	–	–	–
Memory loss	–	+	–
Migraine/Headache	+	+	+
Stroke/Transient ischemic attack	–	–	–
Mood disturbance/Depression	+	+	+
Blurred vision	+	–	–
Aphasia/Speech difficulty	+	–	–
Cognitive decline	–	–	–
Vertigo	–	+	+
Gait imbalance	+	–	+
Numbness/Paresthesia	+	+	+
Bladder symptoms (Urgency/Frequency)	–	–	–
Smoking history	+	+	+

Although the last two patients may be considered as misdiagnosed cases, the clinical findings of myelitis, an extremely rare finding in CADASIL (17), in the first patient were unusual and may raise the question of a concurrent autoimmune response. However, the history of stroke and the extensive leukoencephalopathy on brain MRI should not be overlooked. Although we cannot confirm the same causal mechanism leads to the coexistence of two conditions, CADASIL and MS, the involvement of (systematic) autoimmune mechanisms in some patients with CADASIL is explained (7, 8). A few studies have previously reported a rare occurrence of autoimmune disorders and inflammatory disease process among patients with CADASIL including coexistence of MS confirmed by subsequent response to steroid treatment (18–20). Tanuja et al. reported an interesting case with clinical presentation and confirmed diagnosis of Balo concentric sclerosis (a rare form of MS), who later tested positive for *NOTCH3* mutation (21). They hypothesize that the presence of the *NOTCH3* mutation associated with CADASIL in this patient could raise questions about disease pathogenesis and possible relationships between the two disorders. There are reports of the presence of central nervous system angiitis (22), antiphospholipid (23) or antinuclear (24) antibodies, or autoimmune thrombocytopenia among CADASIL patients (25). Oligoclonal bands in the cerebrospinal fluid, a characteristic finding in MS, have also been reported among CADASIL patients (26). Mutations of the NOTCH protein and components of its signaling pathway have previously been implicated in MS and CADASIL and could play a role in altering the course of the disease (27). It has also been shown that NOTCH3 receptor inhibition can play a role in T-cell response regulation, and selective inhibition of the NOTCH3 receptor may offer a new target in treating autoimmune diseases, including MS (8). Although there is no strong evidence of blood-brain barrier leakage in CADASIL (28, 29), the accumulation of NOTCH3 receptor ectodomain in the vascular wall might potentially increase vascular permeability. Some patients with CADASIL have high protein in CSF that can be evidence of blood-brain barrier dysfunction (30). There are also reports of good response to corticosteroids among patients with CADASIL (18). However, one may argue that partial recovery after a lacunar stroke can be confused with the response to anti-inflammatory treatments. There is also some degree of recovery after an ischemic stroke episode in a patient, which may mislead the physician and give the assumption that the improvement was due to disease-modifying therapy. In any case, it is still questionable if *NOTCH3* mutations can provoke an autoimmune process.

In a recent study, one group prospectively followed 80 CADASIL subjects for a mean period of about 2 years and observed that the extent and mode of progression varied greatly among subjects and that some patients showed a marked and rapid deterioration, whereas others remained stable or even improved. Most patients with worsening disability in this cohort had experienced a new stroke, indicating that recurrent stroke is a major determinant of disability progression in CADASIL (31). Although recurrent stroke is a hallmark of CADASIL, some patients may not have a stroke. A recent study showed that the variability in patients' symptoms could be due to the position of the pathogenic variant in the *NOTCH3* EGFr domain (11). The researchers showed that patients with pathogenic variants in EGFr 7–34 experience a much milder phenotype. The milder form was shown to be associated with a later onset of stroke and more prolonged survival in patients with CADASIL as compared to the more severe form of CADASIL (1). The milder pathogenic variant may explain the late-onset and slow-progressing symptoms in the last case presented above.

Although a family history of stroke and migraine plays a role in diagnosing CADASIL, a negative family history does not preclude the diagnosis, as affected family members may have been misdiagnosed (32) or the patient could be a proband (33). The *de novo* mutations causing CADASIL have previously been reported in literature (33–35). The physicians need to be mindful of the symptoms, such as lacunar infarcts and cognitive impairment occurring among younger patients with no vascular risk factors.

Since CADASIL is a rare disease, it is imperative to raise awareness of its unique clinical condition as well as variation in its clinical presentations. It is crucial that the overlapping symptoms between MS and CADASIL be thoroughly examined to avoid misdiagnosis and treatment complications. The involvement of autoimmune mechanisms in CADASIL and the role of *NOTCH3* mutations in provoking an autoimmune process should be further investigated.

## Data Availability Statement

All datasets generated for this study are included in the article/Supplementary Material.

## Ethics Statement

Written informed consent was obtained from the individuals for the publication of any potentially identifiable images or data included in this article.

## Author Contributions

AK, ME, MM, and RZ reviewed the cases. ME, MM, and RZ provided the critical evaluation of brain images. AK, RZ, and JL summarized the findings. AK drafted the manuscript. RZ and VA contributed to the writing of different sections. All authors read and approved the final manuscript.

## Conflict of Interest

The authors declare that the research was conducted in the absence of any commercial or financial relationships that could be construed as a potential conflict of interest.
