# Supplementary material for: CADASIL vs. Multiple Sclerosis: Is It Misdiagnosis or Concomitant? A Case Series
Source: Front Neurol. 2020 Sep 4;11:860. doi: 10.3389/fneur.2020.00860 (PMC7500095; doi:10.3389/fneur.2020.00860)
Supplement: Supplementary file 1 [file Data_Sheet_1.PDF]

CARE case report guidelines

CARE Checklist of information to include when writing a case report

(cc) BY-NC-ND

Topic	Item	Checklist item description	Reported on Line
Title	1	The diagnosis or intervention of primary focus followed by the words "case report"	1
Key Words	2	2 to 5 key words that identify diagnoses or interventions in this case report, including "case report"	38
Abstract (no references)	3a	Introduction: What is unique about this case and what does it add to the scientific literature?	17-21
	3b	Main symptoms and/or important clinical findings	22-32
	3c	The main diagnoses, therapeutic interventions, and outcomes	22-32
	3d	Conclusion—What is the main "take-away" lesson(s) from this case?	33-36
Introduction	4	One or two paragraphs summarizing why this case is unique (**may include references**)	50-52
Patient Information	5a	De-identified patient specific information.	52-54
	5b	Primary concerns and symptoms of the patient.	58,93,107
	5c	Medical, family, and psycho-social history including relevant genetic information	63, 99, 111
	5d	Relevant past interventions with outcomes	67,71,78,95,115
Clinical Findings	6	Describe significant physical examination (PE) and important clinical findings.	67,71,78,95,115
Timeline	7	Historical and current information from this episode of care organized as a timeline	65-84
Diagnostic Assessment	8a	Diagnostic testing (such as PE, laboratory testing, imaging, surveys).	74-76
	8b	Diagnostic challenges (such as access to testing, financial, or cultural)	NA
	8c	Diagnosis (including other diagnoses considered)	77,100, 106
	8d	Prognosis (such as staging in oncology) where applicable	NA
Therapeutic Intervention	9a	Types of therapeutic intervention (such as pharmacologic, surgical, preventive, self-care)	72, 117
	9b	Administration of therapeutic intervention (such as dosage, strength, duration)	72, 117
	9c	Changes in therapeutic intervention (with rationale)	83,101,118
Follow-up and Outcomes	10a	Clinician and patient-assessed outcomes (if available)	73,94,115
	10b	Important follow-up diagnostic and other test results	80, 101, 112,
	10c	Intervention adherence and tolerability (How was this assessed?)	73
	10d	Adverse and unanticipated events	NA
Discussion	11a	A scientific discussion of the strengths AND limitations associated with this case report	129
	11b	Discussion of the relevant medical literature **with references**.	135
	11c	The scientific rationale for any conclusions (including assessment of possible causes)	165
	11d	The primary "take-away" lessons of this case report (without references) in a one paragraph conclusion	166
Patient Perspective	12	The patient should share their perspective in one to two paragraphs on the treatment(s) they received	85
Informed Consent	13	Did the patient give informed consent? Please provide if requested	**Yes** x ■ **No** ■
